# Accuracy and consistency of intensity-based deformable image registration in 4DCT for tumor motion estimation in liver radiotherapy planning

**DOI:** 10.1371/journal.pone.0271064

**Published:** 2022-07-08

**Authors:** José D. Tascón-Vidarte, Line Bjerregaard Stick, Mirjana Josipovic, Signe Risum, Julien Jomier, Kenny Erleben, Ivan Richter Vogelius, Sune Darkner

**Affiliations:** 1 Department of Computer Science, University of Copenhagen, Copenhagen, Denmark; 2 Department of Oncology, Copenhagen, Denmark; 3 Kitware SAS, Villeurbanne, France; University Hospital Zurich, SWITZERLAND

## Abstract

We investigate the accuracy of intensity-based deformable image registration (DIR) for tumor localization in liver stereotactic body radiotherapy (SBRT). We included 4DCT scans to capture the breathing motion of eight patients receiving SBRT for liver metastases within a retrospective clinical study. Each patient had three fiducial markers implanted. The liver and the tumor were delineated in the mid-ventilation phase, and their positions in the other phases were estimated with deformable image registration. We tested referenced and sequential registrations strategies. The fiducial markers were the gold standard to evaluate registration accuracy. The registration errors related to measured versus estimated fiducial markers showed a mean value less than 1.6*mm*. The positions of some fiducial markers appeared not stable on the 4DCT throughout the respiratory phases. Markers’ center of mass tends to be a more reliable measurement. Distance errors of tumor location based on registration versus markers center of mass were less than 2*mm*. There were no statistically significant differences between the reference and the sequential registration, i.e., consistency and errors were comparable to resolution errors. We demonstrated that intensity-based DIR is accurate up to resolution level for locating the tumor in the liver during breathing motion.

## Introduction

Stereotactic body radiotherapy (SBRT) is one of the treatment modalities used for managing tumors in the liver [1]. The SBRT delivery is, however, affected by the respiratory motion of the target [2], resulting in potential underdosing of the target and/or overdosing to organs-at-risk [3–5]. Different active methods such as breath-hold, shallow breathing, and abdominal compression have been proposed to control or reduce the effect of the breathing motion [6, 7]. Another approach is a passive compensation method to explicitly determine breathing cycle margins and compute an internal target volume (ITV) [8] that is included in the planning target volume (PTV) estimation [9, 10]. Nonetheless and regardless of the method, breathing motion has to be accounted for during planning and treatment.

The feasibility of using deformable image registration (DIR) to estimate breathing motion and tumor positions in the liver has been demonstrated [11], aiding the evaluation of the positional uncertainties in both target and risk organs, induced by the respiration [12]. Although DIR has been increasingly used in radiotherapy [13, 14], its accuracy is difficult to quantify and is considered as a non-trivial task [15]. This difficulty is caused by the lack of ground truth data. Several DIR algorithms have been developed, but they have been mostly evaluated on images from the thorax [15] which have manually annotated landmarks [16, 17]. In contrast, the accuracy in low contrast organs (abdominal area) has only been studied sparsely [12].

Liver tumors are often not visible without admission of intravenous contrast on either CT or cone-beam CT (CBCT) [18, 19]. Therefore gold fiducial markers can be implanted in the tumor vicinity [20] and used as a surrogate of the tumor position during SBRT [21]. Image guidance with fiducial markers is reported as a more accurate method compared to image guidance with the liver contour or diaphragm position [22]. Therefore, we use fiducial markers to evaluate tumor registration accuracy in our study.

Regarding the DIR algorithms on the liver, the finite element model-based DIR proposed by Brock et al. [23] is the only algorithm to be extensively evaluated in CT with low contrast organs [11, 24, 25]. The MIDRAS study [26] compared several algorithms for the abdomen and the liver with only one 4DCT scan. Finite element model-based DIR methods seem to perform slightly better than intensity-based algorithms for the liver. The drawback is that one patient is not representative of the general performance of the algorithms. Furthermore, the intensity-based methods here only included demons [27] and B-splines [28, 29] algorithms and not the modern large diffeomorphic metric mapping algorithms [30, 31]. An advantage of intensity-based DIR is that it computes registration without prior information or preprocessing. In contrast to the finite element model-based DIR that requires organ segmentation, surface mesh conversion, boundary conditions, and manual parametrization of material properties [23]. We consider that a modern intensity-based algorithm requires a proper evaluation of registration accuracy for liver radiotherapy due to its implicit automation capabilities.

In this paper, we aim to demonstrate that the use of intensity-based DIR is accurate in estimating the position of liver tumors in low contrast images. We applied DIR to respiratory correlated 4DCT and used implanted fiducial markers as a surrogate of tumor locations.

## Materials and methods

### Patients

This study was based on data from eight patients treated with SBRT for metastases in the liver at Rigshospitalet (Copenhagen, Denmark) between March 2018 and September 2019. The patients were selected from a group of patients, participating in a prospective clinical protocol investigating liver SBRT in breath-hold (approved by The Committees on Health Research Ethics in the Capital Region of Denmark; approval nr. H-17033786). All patients signed an informed consent before the enrollment in the study. This consent included use of their imaging data for research purposes. See details on patient selection in S1 Appendix.

### Image acquisition

Respiratory correlated 4DCT with intravenous contrast injection was performed for all patients on a SOMATOM Definition AS scanner (Siemens Healthineers, Germany). 4DCT image data were phase-sorted into ten phase bins throughout a respiratory cycle based on an external respiratory signal monitored with Real-Time Position Management (RPM, Varian Medical Systems, Palo Alto, CA). The slice separation in each phase of the 4DCT was 2*mm*. The image resolution in each slice was 512 × 512 pixels and a pixel size of 0.98 × 0.98 mm.

In addition, MR scans in deep inspiration breath-hold without visual guidance were performed for all patients to achieve better visualization of the tumor. The MR images (T1 VIBE with intravenous contrast, T1 FL2D, T2 HASTE, TrueFISP) were acquired in a 1.5T SIEMENS MAGNETOM Avanto scanner (Siemens Healthineers, Germany).

### Delineations

**Fig 1:**
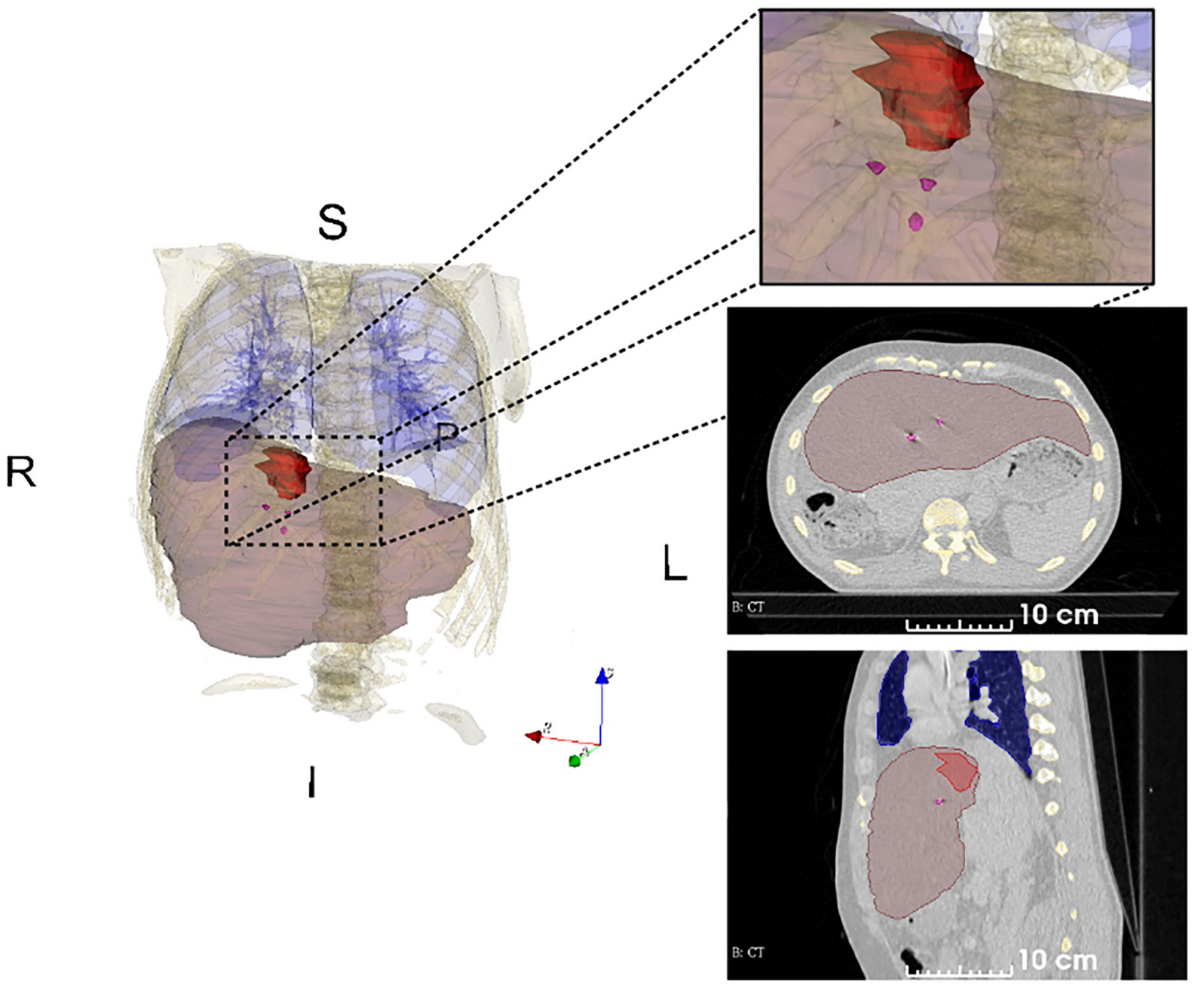
A single CT image of the 4DCT with organ delineations. The image corresponds to mid-ventilation phase of patient 2. Delineations of liver (translucent brown), gross tumor volume (red) and fiducial markers (magenta). The fiducial markers were implanted near the tumor. The delineated volumes were used to compute the centroids of the liver, the gross tumor volume (GTV) and the fiducial markers.

The mid-ventilation phase of the 4DCT was determined based on fiducial marker (tumor) motion for each patient and used for treatment planning [32]. Further details of treatment are out of the scope of the paper. The mid-ventilation phase was rigidly registered (in 6D) with the MR scan focusing on the tumor area to guide delineation of the tumor. This rigid registration is performed manually using the fiducial markers locations. The Gross Tumor Volume (GTV) was delineated on mid-ventilation phase by a senior radiologist and approved by an independent senior oncologist. Risk organs, including the liver, were also delineated. Fig 1 shows the mid-ventilation phase for patient 2.

### Fiducial markers

Three fiducial markers were implanted percutaneously near the tumor in each patient using ultrasound guidance. The fiducial markers were placed within one week prior to the imaging for treatment planning. Goldlock fiducial markers (cylinder with star-shaped cross-section, 1 x 3 mm, Beampoint AB, Sweden) were implanted in the first five patients and Gold Anchor fiducial markers (cylinder with multiple cut-outs, 0.4 x 10 mm, Naslund Medical AB, Sweden) were implanted in the latter three patients.

The centroid position of each fiducial marker was calculated automatically in each phase and chosen as its ground truth locations. The automatic procedure consisted of three steps. First, a threshold of 500 Hounsfield units was applied to the images. Second, the fiducial markers were delineated in the axial plane in all 4DCT phases. Third, the delineations were used to create a volume of the fiducial marker and then the centroid is computed. The accuracy of this method was compared to the manual method for locating the center of the fiducial markers [33]. The comparison was made with all 4DCT phases of a single patient and the values were approximately the same (differences <0.1*mm*). The uncertainty of the fiducial markers centroids were expected to be greater in the SI plane due to the 2 mm slice separation.

The fiducial markers center of mass (COM) are calculated for each patient as the average position of the three fiducial markers. From here, we will refer to this measurement simply as markers COM, while ‘fiducial markers’ refer to the (individual) positions of the markers centroids.

### Registration algorithm

The publicly available Symmetric Image Normalization algorithm (SyN) [31] was used as the image registration algorithm, being one of the best publicly available algorithms for DIR [12]. Briefly, the SyN algorithm uses Cross Correlation (CC) as similarity metric and the L2 norm of the velocity field as regularization. The transformation or displacement field is then computed by integration of the differential equation that relates velocity with displacement. The mathematical details are exposed in S2 Appendix.

**Fig 2:**
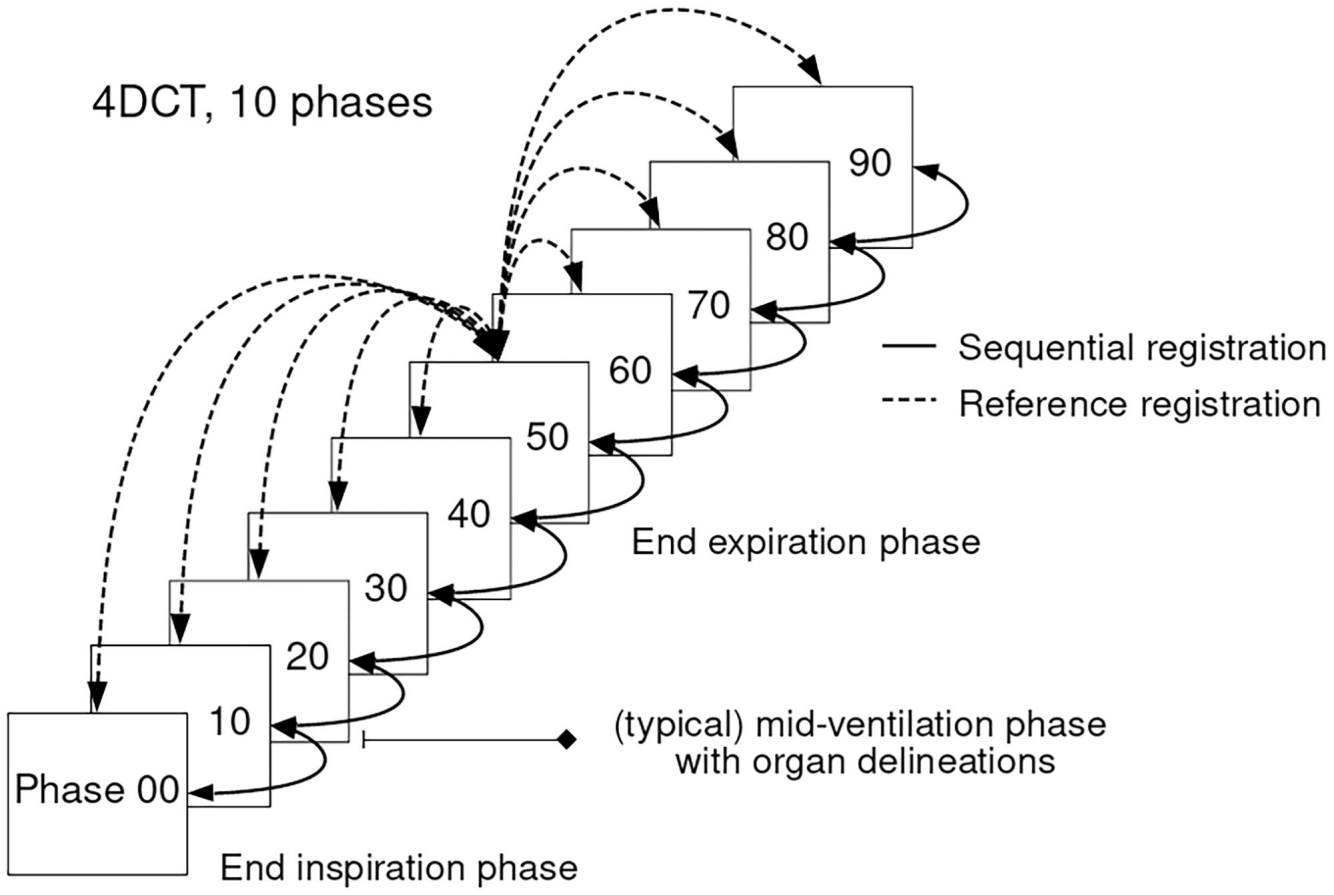
4DCT scan with a representation of reference and sequential registration. Delineations of the liver and gross tumor volume (GTV) are propagated from mid-ventilation phase to the other phases. The 50% phase corresponds (approximately) to end expiration and chosen as the reference image.

In this work, the DIR was computed in two ways, depicted in Fig 2. The first method used a single image as reference and the remaining images were subsequently registered to this reference image. The reference image was chosen as the 50% phase of the 4DCT which approximately corresponded to the end of expiration. The second method registered all the images sequentially. For the sake of simplicity, we will refer to the methods as reference and sequential registration through out this manuscript. We compared the registration errors of the two methods in order to investigate if sequential registration introduces a drift error due to composition of the estimated deformations.

The liver and GTV delineations were propagated to the other phases of the 4DCT with the DIR algorithm using only the sequential approach. The appropriated transformation was chosen as the lowest number of composition of deformations from the mid-ventilation phase to the other phases. After DIR, a volume was created in each phase for each patient to compute the center of mass (COM) of the tumor and the liver. From here, we will refer to this positions as liver COM and GTV COM. This methodology is referred as segmentation based on DIR or contour propagration [34]. The accuracy of this estimation is therefore related to the DIR error.

The DIR algorithm is voxel intensity-based. In this scenario, the fiducial markers became a problematic surrogate for registration, since they create high intensity values around their location [35]. In order to overcome this, image inpainting was applied to remove the high intensity voxels.

### Image inpainting

The image inpainting was performed with the algorithm proposed by Telea et al. [36]. For extended details of the inpainting see S3 Appendix. After all the 4DCT images are inpainted, the DIR algorithm is applied to all the patients. Only the reference registration approach is used here. The measured fiducial markers coordinates are still used for computing the registration errors. The registration of the inpainted images was compared to the registration without the inpainting. In case of similar results we could conclude that the registration was not driven by the high intensities of the fiducial markers.

### Distance metrics

**Fig 3:**
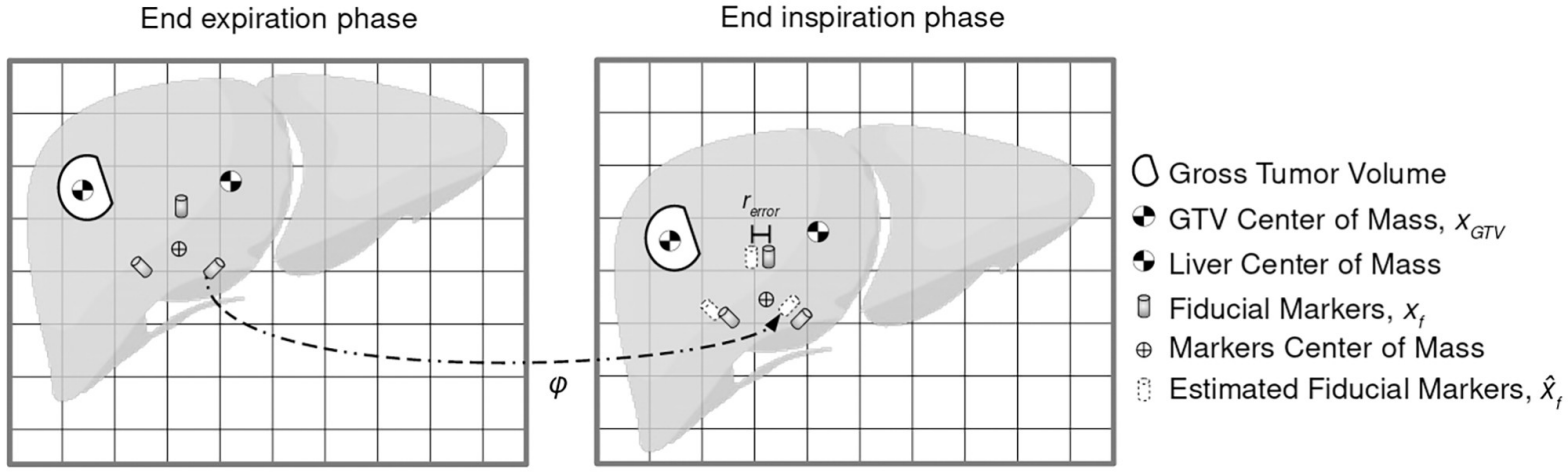
Landmarks used to compute registration and distance metrics. Center of mass (COM) are calculated for fiducial markers, gross tumor volume (GTV) and liver. GTV and liver volumes are estimated in all phases via contour propagation based on DIR from the mid-ventilation phase. Estimated fiducial markers’ positions are computed with the transformation *φ* found by registration. The registration error (*r*_*error*_) from Eq (1) is illustrated here as the euclidean distance of estimated fiducial markers’ position versus measured fiducial markers’ position.

The fiducial markers were used as the direct measurement to evaluate the accuracy of image registration. Two distance metrics were used: the first one for DIR evaluation and the second one to verify the relative distance variations of markers, tumor and liver. Fig 3 presents all the points used to compute the distance metrics.

The first metric was the registration error calculated as the euclidean distance between the ground truth positions of the fiducial markers and the estimated positions of the fiducial markers. The ground truth coordinate was a point *x*_*f*, *j*_, where the subscripts *f* and *j* refer to the particular fiducial marker and the image phase respectively. The transformation found by registration *φ*_*j*_ was applied to the fiducial markers in the reference phase *x*_*f*,ref_, producing the estimated fiducial markers ${\hat{x}}_{f,j}=\varphi_{j}\circ x_{f,\text{ref}}$. The metric is defined as:

$$\begin{array}{c} r_{\text{error}}\equiv \|x_{f,j}-\underset{{\hat{x}}_{f,j}}{\underset{︸}{\varphi_{j}\circ x_{f,\text{ref}}}}\| \end{array}$$ (1)

The registration errors are calculated for the reference and sequential registration algorithms, as well as for images registered with and without inpainting. The statistical tool used to compared the data is the Kolmogorov-Smirnov test [37].

The second metric was the relative distance variation. In this case, the distance between two points was compared to check if the distance was the same across the phases. The metric was calculated between the fiducial markers, between the GTV COM and the fiducial markers, between the GTV COM and the markers COM, and between the GTV COM and the liver COM. As an example, the relative distance of the fiducial marker 1 and fiducial marker 2 is presented here, as:

$$\begin{array}{c} d\equiv \|(x_{f2,j}-x_{f1,j})\|-\|(x_{f2,\text{ref}}-x_{f1,\text{ref}})\| \end{array}$$ (2)

## Results

### Breathing motion

Table 1 summarizes breathing motion characteristics of the patients. The average displacement of the fiducial markers between expiration and inspiration for all the patients was *μ* = 7.9 mm with a standard deviation of *σ* = 2.9 mm. The fiducial markers distances to the GTV COM ranged from 17 to 55 mm, with mean value *μ* = 32 mm and standard deviation of *σ* = 10 mm.

**Table 1:** Summary of patients information.

	Patient	1	2	3	4	5	6	7	8
Tumor location	Geometric	I-A-L	S-A-R	S-P-R	S-P-L	I-P-R	S-A-L	S-P-L	I-P-L
Breathing cycle [s]	Mean	2.8	3.5	3.0	4.1	5.8	4.0	5.3	3.3
Tumor displacement [mm]	LR	1.2	0.9	3.6	2.5	2.5	1.3	0.4	0.8
	AP	1.3	0.6	3.4	0.5	3.3	1.8	2.3	2.9
	SI	5.1	7.8	10.2	6.3	12.6	3.7	8.2	7.9
	3D	5.4	7.9	11.2	6.7	13.3	4.0	8.5	8.3
Distance GTV COM to [mm]	Marker 1	20.6	19.0	30.8	43.5	33.8	49.0	25.0	30.8
	Marker 2	29.5	24.0	41.1	41.5	28.3	33.1	21.1	25.7
	Marker 3	17.9	41.6	18.0	44.6	54.9	31.5	36.9	40.8
	Marker COM	22.3	40.3	26.6	32.2	26.1	18.1	38.7	29.9
Distance between markers [mm]	Marker 1—2	12.0	15.9	14.1	30.4	25.3	19.6	23.3	21.3
	Marker 2—3	16.6	29.5	21.6	35.7	20.1	15.2	17.3	31.7
	Marker 3—1	29.2	58.9	31.8	14.2	41.9	40.8	19	43.7
GTV [cm3]	Mid-ventilation	7.6	29.0	29.3	11.6	2.4	4.0	4.5	64.8
	Mean	7.4	29.7	28.8	11.4	2.5	4.2	4.4	65.4
	CV [%]	0.5	1.2	1.7	2.4	0.1	0.4	0.9	1.0
Liver volume [cm3]	Mid-ventilation	1904	2324	1343	1458	912	2156	1744	1596
	Mean	1908	2321	1343	1458	903	2172	1722	1594
	CV [%]	7.6	1.4	3.7	5.1	12.5	10.1	10.7	3.5

Table notes: Tumor location is the geometric octant of where the tumor is with regards to the liver COM. The abbreviations used correspond to Superior-Inferior, Anterior-Posterior and Left-Right. Breathing cycle time was determined from the external respiratory signal, acquired during the 4DCT scan. Tumor displacements are computed using the markers COM as surrogate of the tumor. Distance of GTV COM to fiducial markers and between markers are measured in the mid-ventilation phase. GTV and liver volumes are estimated for every phase based on DIR. COM = center of mass, CV = coefficient of variation.

The sequential registration was used to estimate volume variations in the liver and the GTV calculated on every phase. The baseline volume measured in mid-ventilation phase is shown in Table 1. The patients in this study had a liver volume coefficient of variation (CV) of 6.5% in average and a maximum of 12.5% (patient 5). The GTV coefficient of variation (CV) was on average 1.0% and a maximum of 2.4% (patient 4). The registration indicated that compressibility in general was low for the liver, and was higher for the liver tissue compared to the tumor tissue.

### Accuracy and consistency of registration

We tested the registration algorithm with end expiration and end inspiration phases (the 50% phase and 0% phase were used for all patients) where the maximum displacement occurs. The algorithm was parametrized to achieve convergence in this scenario. For further details see S4 Appendix. The average time of the DIR algorithm was 170.5 minutes (per registration), running in a workstation with two processors Intel Xeon Silver 4110 (8 cores, 16 threads each cpu).

**Fig 4:**
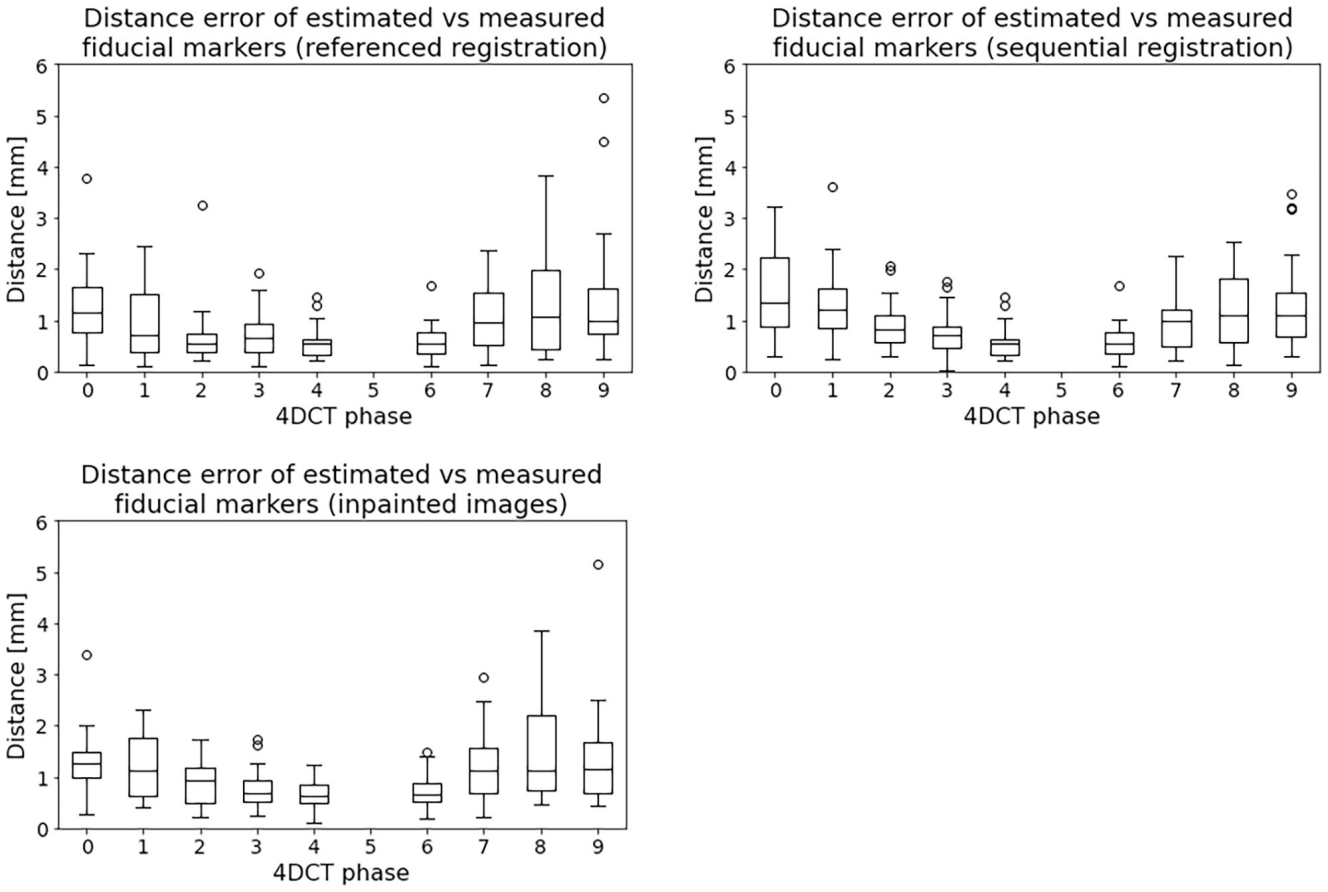
Distance metric (Eq (1)) of fiducial markers for all the patients computed with the reference (top-left) and the sequential registration (top-right). End expiration was chosen as reference of measurements, i.e. 50% phase. The top figures show the distance between the estimated and the measured fiducial markers in all phases for all patients. The estimated fiducial markers are the transformed markers from the reference phase to the corresponding 4DCT phase using the transformation found with registration. The bottom figure depicts the registration errors for the inpainted images. Only the reference registration approach is tested for the inpainted images.

We tested the registration errors (Eq (1)) in all patients and all markers per phase (see Fig 4). The registration errors of the estimated fiducial markers versus measured fiducial markers had a maximum mean value of 1.4*mm* for the referenced registration and 1.6*mm* for the sequential registration, shown in Fig 4 top-left panel and Fig 4 top-right panel respectively. The corresponding median values were up to 1.2*mm* and 1.4*mm* respectively. An increased mean and standard deviation was noted for the images further away of the reference phase for both reference and sequential registration. There were no statistically significant differences per phase related to the registration method. See more details in S5 Appendix.

The maximum registration errors were 5.3*mm* and 3.7*mm* for the referenced registration and the sequential registration respectively. More details are presented in S6 Appendix. The registration errors always have at least one fiducial marker with an error lower than 2*mm* for each patient.

The inpainted images were tested only for the referenced registration case with the registration error metric and we found no statistically significant differences compared to the original images, see Fig 4 bottom panel and S5 Appendix.

As we observed with the registration errors that the average maximum obtained was equivalent or lower than the resolution in the axial slices. Since the reference and the sequential registration did not produce statistically significant differences this indicates a consistency in the registration method to accurately align the images.

### Distance variations of markers, GTV and liver

**Fig 5:**
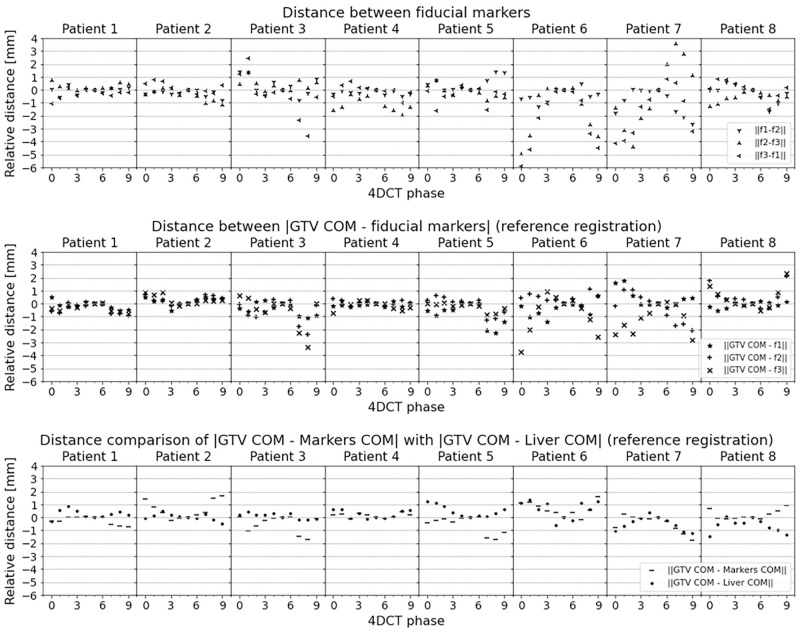
Comparisons of markers distance stability. The distances within each phase are measured relatively to the base distance measured at reference phase (50% phase). The top panel shows the distance between the markers. For some patients the marker to marker distance differ in the breathing cycle up to 6*mm*. This clearly demonstrates that individual markers are problematic surrogates of the tumor in the liver. The middle panel shows the distance between the GTV COM and each fiducial marker. Some of the markers change their distance with the tumor up to 4*mm*. The bottom panel depicts the relative distance of the GTV COM with the liver COM. GTV COM and liver COM are estimated with deformable image registration. Errors are up to 1.2*mm*.

Fig 5 shows the distance variations of markers, GTV and liver for each patient. The corresponding distances were computed similar to Eq (2). For reference values see Table 1. Fig 5 top panel depicts the euclidean distance between the markers for each phase. The distance between markers varied up to 6 mm.

Fig 5 middle panel presents the relative distance variations of GTV COM and fiducial markers per patient. Since the GTV COM is estimated with registration, this particular metric is related to the registration error. It can be seen that when a high distance variation between fiducial markers occur, it corresponds to a high variation of GTV COM and fiducial markers, and therefore, a high registration error. This is also observed in S4 Fig of S6 Appendix. This pattern is noticed for patient 3, 6 and 7. As an example, if we analyze fiducial marker 3 of patient 6 at 0% phase, the GTV COM to fiducial marker distance is close to 4*mm*. This error is justified in Fig 5 top panel, where there are distance variations of fiducial marker 3 with the other markers. We could confirm an uncertainty in the measurement of fiducial marker 3. Individual analysis could be made for the others fiducial markers. In order to avoid individual fiducial marker errors the markers COM is recommended.

For patient 8 a high registration error of marker 1 in phase 90% is related to the position of the marker close to the edge of the liver. For this case, image qualitative verification is a valid tool of assessment, as suggested in the AAPM TG-132 report on image registration [14].

Fig 5 bottom panel shows the relative distance variations between GTV COM and markers COM, and between GTV COM and liver COM. The GTV COM—liver COM relative distance variation was computed with registration. GTV COM to markers COM distance variations were up to 2*mm*. Assuming that the real position of the GTV was followed by the marker COM the accuracy of registration was as good as the resolution of the image. GTV COM to liver COM distance errors were lower than 1.2*mm*. Both COMs were computed from registration and they followed the same motion pattern.

## Discussion

We have demonstrated that the application of a publicly available SyN deformable image registration algorithm on a specific dataset, acquired for liver SBRT was suitable for liver and GTV localization. We investigated the accuracy of estimated versus measured fiducial markers’ positions and shown that the mean error was less than 1.6*mm*. This suggested that intensity-based DIR is accurate for liver radiotherapy (< 2 − 3*mm* [14]).

We found no statistically significant differences between registering the images sequentially or to a reference. These results are in accordance to the results found by Boldea et al. for lungs [38]. In contrast to rigid motion problems where a drift is presented to sequentially registered images [39], we found that DIR errors in liver 4DCT scans were of smaller magnitude than resolution errors.

It could be argued that the high intensity artifacts created by the fiducial markers influenced the convergence and accuracy of the registration algorithm. In order to investigate this hypothesis, we performed a test with the same algorithm on the same images where inpainting was applied to remove the fiducial markers. Only the reference registration is tested. No statistically significant difference was found from the results obtained using the original images (without inpainting). This also suggest that the DIR is driven by the whole abdomen (liver) intensities values.

We expose the other capabilities of DIR applied to the liver. These are the estimation of liver and tumor volumes in the breathing cycle. The liver tissue is not highly compressible, but some deformation is expected [40]. Another capability is contour propagation. Contour propagation with DIR have been evaluated before for lung and head and neck tumors [34]. To the best of our knowledge, this is the first time that is evaluated for the liver. Although, this is not validated with overlapping metrics, the visual quality and the registration errors suggested succesful results.

We always evaluated the registration accuracy with features or points, i.e. fiducial markers. Other metrics such as image similarity or volume overlapping are less recommended in DIR [41]. The registration errors always had at least one fiducial marker with an error lower than 2*mm* for each patient. Some of the maximum values in registration errors could be seen as outliers, and may be due to uncertainties in the measurement of individual fiducial markers.

Analysis of the individual results of each patient in Fig 5 revealed that the errors came from the fiducial markers positions that seemed unstable between the respiratory phases. These variations could be related to two factors, measuring errors or liver compression/deformation. These relatively high values demonstrated that a single fiducial marker cannot be a reliable surrogate for tumor positioning. One explanation of a measuring error could be related to an artefact, induced during 4DCT reconstruction due to an unstable respiratory pattern [42]. The other reason is that compression/deformation inherently change the distance between markers. This behavior may induce errors in the routine location of the tumor inside the liver which is dependent on the markers. We propose then the use of a more consistent reference for locating the GTV COM with the liver COM computed with registration. This reference shows a maximum relative error of 1.2 mm inter-phases. Therefore, the approach taken here could reduce potential errors.

Compared to some of the previous studies related to fiducial markers in liver radiotherapy [20, 21, 33, 43–45], to the best of the author’s knowledge, this is the first study reporting variations in marker to marker distances between phases. This factor points out to additional induced errors with fiducial markers as a surrogate due to measurement errors or to liver deformations. Similarly to the previous studies, we also confirmed that the fiducial markers COM is a better surrogate of the liver compared to single fiducial markers. It is also expected that some uncertainty exists in the markers COM calculation. As suggested by Wunderink et al. [20], the fiducial markers should be implanted near and surrounding the tumor. In our study the fiducial markers were closely located between 17*mm* and 55*mm*. We used two types of fiducials in our study. We observed that the generated imaging artifacts are similar in intensity and volume. Therefore we consider for our case that the COM estimation is equivalent and fiducial type independent.

One of the limitations of our study is the small number of patients. A larger number of patients would allow pinpointing of situations with lower accuracy. Furthermore, including more patients would strengthen the results, however for a proof of principle, the included number seems sufficient, since a wide range of tumour positions within the liver and respiratory motion amplitudes was represented in our patient material. Another drawback prior to a potential clinical implementation is current DIR computational time. This may be overcome in a near future with SyN algorithm implementations on GPU [46] or emerging deep learning DIR methods [47] where results are obtained in a couple of minutes or even seconds respectively.

## Conclusion

We have demonstrated that intensity-based DIR is accurate for liver radiotherapy. Furthermore, we found no statistically significant differences between the images sequentially or referenced DIR methods. We propose a GTV localization strategy on 4DCT using DIR, that is consistent with GTV localization based on fiducial markers’ COM with less variability than what is seen between individual markers. The DIR algorithm using the liver COM as a reference to locate the GTV, resulted in our small patient cohort in a maximum error of 1.2*mm*. Such accuracy also seems adequate for radiotherapy.

## Supporting information

S1 Appendix (PDF)

S2 Appendix (PDF)

S3 Appendix (PDF)

S4 Appendix (PDF)

S5 Appendix (PDF)

S6 Appendix (PDF)

S7 Appendix (PDF)

S1 Data (XLSX)
