# Supplementary material for: Accuracy and consistency of intensity-based deformable image registration in 4DCT for tumor motion estimation in liver radiotherapy planning
Source: PLoS One. 2022 Jul 8;17(7):e0271064. doi: 10.1371/journal.pone.0271064 (PMC9269460; doi:10.1371/journal.pone.0271064)
Supplement: S1 Appendix — (PDF) [file pone.0271064.s001.pdf]

S1 Appendix

Patient Selection

The patients were selected within a breath-hold study of SBRT for metastases. The patients were treated at Rigshospitalet, Copenhagen, Denmark. The initial 21 patients were reduced to 8 patients based on fiducial markers implanted, image quality and regular breathing patterns. See details on patient selection in S1 Fig.

As a part of the study protocol the patients were also imaged using CT in three deep inspiration breath-holds and three expiration breath-hold using the RPM system with visual feedback to obtain consistent breath-hold levels.

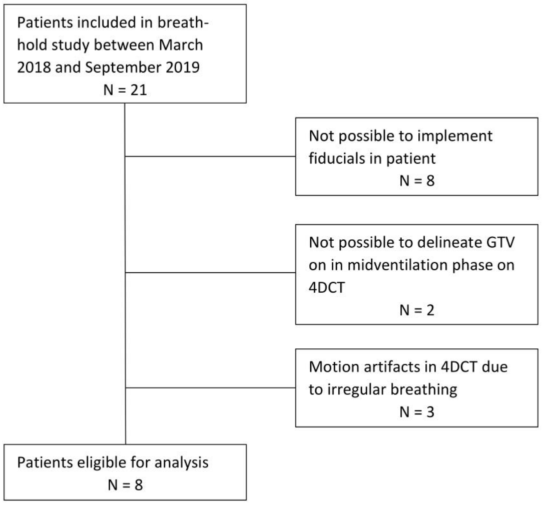

S1 Fig. Flow chart of patient selection. A total of 8 patients with fiducial markers were selected to test the accuracy of registration to locate the tumor..
