# Supplementary material for: Accuracy and consistency of intensity-based deformable image registration in 4DCT for tumor motion estimation in liver radiotherapy planning
Source: PLoS One. 2022 Jul 8;17(7):e0271064. doi: 10.1371/journal.pone.0271064 (PMC9269460; doi:10.1371/journal.pone.0271064)
Supplement: S2 Appendix — (PDF) [file pone.0271064.s002.pdf]

S2 Appendix

Registration Algorithm

Image registration is the process of estimating the transformation between two images in order to align them in a single coordinate system. The algorithm is called deformable image registration (DIR) when the images need to be transformed in a deformable manner, typically with a deformation field. This is the case of images with internal organs of the human body acquired during breathing motion.

DIR is expressed as computing the diffeomorphic mapping $\varphi(x)$ of the moving image I_1 into the reference image I_0. The registration problem can be posed as a cost- or energy optimization problem involving a similarity metric E_M and a regularization term E_R, such as:

$$\varphi^*(x) \equiv \underset{\varphi(x)}{\arg\min} \quad \underbrace{E_M(I_0, I_1 \circ \varphi(x)) + E_R(\varphi(x))}_{\equiv E(\varphi(x))} \tag{3}$$

Where:

$\varphi(x) : \Omega_0 \to \Omega_1$, $x \in \Omega$, $\Omega \subset \Re^d$,

$I_j : \Omega_j \to \Re, \forall j \in \{0, 1\}$.

Avants et al. [1] introduce Symmetric Image Normalization (SyN) a DIR algorithm. The algorithm is available with ANTs toolbox [2] and supported by ITK toolbox [3].

SyN algorithm uses cross correlation as the similarity metric E_M defined as:

$$E_M(I_0, I_1 \circ \varphi(x)) = CC(I_0, I_1, \varphi(x)) \equiv \frac{\left(\sum\limits_{x \epsilon \Omega_0}^{n} (I_0(x) - \bar{I}_0) \cdot (I_1 \circ \varphi(x) - \bar{I}_1)\right)^2}{\sum\limits_{x \epsilon \Omega_0}^{n} (I_0(x) - \bar{I}_0)^2 \cdot \sum\limits_{x \epsilon \Omega_0}^{n} (I_1 \circ \varphi(x) - \bar{I}_1)^2} \tag{4}$$

The regularization E_R is defined as the Sobolev norm of the velocity field v_t, similar to the term proposed by Beg et al. [4]. The velocity field is related to the deformation field $\varphi(x)$ with the ordinary differential equation $\frac{d\phi_t(x)}{dt} = v_t(\phi_t(x), t)$, with solution $\varphi(x) = \phi_0 + \int_0^1 v_t(\phi_t(x))dt$. In order to address consistency and reduce computation time, SyN algorithm divide the deformation field in two, such as: $\varphi(x) = \phi_1 \circ {\phi_2}^{-1}(x, t)$. Consequently, the regularization term involves two velocity fields. The mathematical setup is expressed as follows:

$$E_R(\varphi(x)) = \int_0^1 \parallel v_t \parallel_L^2 \, dt = \int_{t=0}^{0.5} \left(\parallel v_1(x,t) \parallel_L^2 + \parallel v_2(x,t) \parallel_L^2\right) dt \tag{5}$$

Where:

$L = a\nabla + bId$, is a linear differential operator that induces regularity.

The optimization in SyN is described by the minimization of the energy equation:

$$\varphi^*(x) \equiv \underset{\varphi(x)}{\arg\min} \underbrace{\int_{t=0}^{0.5} \left(\parallel v_1(x,t) \parallel_L^2 + \parallel v_2(x,t) \parallel_L^2\right) dt + \int_\Omega CC(I_0, I_1, \varphi(x)) d\Omega}_{\equiv E(\varphi(x))} \tag{6}$$

References

1. Avants BB, Epstein CL, Grossman M, Gee JC. Symmetric diffeomorphic image registration with cross-correlation: evaluating automated labeling of elderly and neurodegenerative brain. Medical image analysis. 2008;12(1):26–41.

2. ANTs. Advanced Normalization Tools; 2021. Web Page: http://stnava.github.io/ANTs/.

3. ITK. Insight Segmentation and Registration Toolkit; 2021. Web Page: https://itk.org/.

4. Beg MF, Miller MI, Trouvé A, Younes L. Computing large deformation metric mappings via geodesic flows of diffeomorphisms. International journal of computer vision. 2005;61(2):139–157.
