# Supplementary material for: Accuracy and consistency of intensity-based deformable image registration in 4DCT for tumor motion estimation in liver radiotherapy planning
Source: PLoS One. 2022 Jul 8;17(7):e0271064. doi: 10.1371/journal.pone.0271064 (PMC9269460; doi:10.1371/journal.pone.0271064)
Supplement: S3 Appendix — (PDF) [file pone.0271064.s003.pdf]

S3 Appendix

Inpainting

The image inpainting algorithm proposed by Telea et. al. [1] is publicly available in OpenCV [2]. The fiducial marker centroid is used as the center point of the region where the inpainting is applied. A square area of 30 by 30 pixels is removed of the axial slices. The region is removed for a total of three slices around the marker. S2 Fig depicts the inpainting of a patient. We used a square area instead of another shape (for instance, a circle) because typical inpainting algorihtms are designed to work with bounding boxes (rectangles).

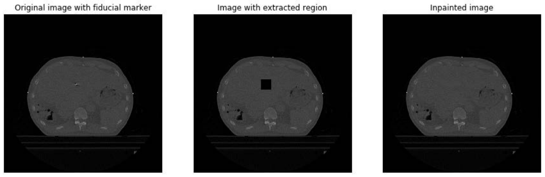

S2 Fig. Example of image inpainting in one axial slice. The inpainted area is a square of 30 pixels around the fiducial marker centroid.

References

1. Telea A. An image inpainting technique based on the fast marching method. Journal of graphics tools. 2004;9(1):23–34.

2. OpenCV. Open Source Computer Vision Library; 2021. Web Page: https://opencv.org/.
