# Supplementary material for: Accuracy and consistency of intensity-based deformable image registration in 4DCT for tumor motion estimation in liver radiotherapy planning
Source: PLoS One. 2022 Jul 8;17(7):e0271064. doi: 10.1371/journal.pone.0271064 (PMC9269460; doi:10.1371/journal.pone.0271064)
Supplement: S4 Appendix — (PDF) [file pone.0271064.s004.pdf]

S4 Appendix

Registration Parametrization

The algorithm is setup in a multi-resolution framework. We computed the distance error per iteration to compare with the cross correlation (CC) metric. S3 Fig depicts the registration for one patient with a maximum of 300 iterations per level. In the multi-resolution scheme it was observed that the intensity metric could induce some overfitting behavior to the metric position of the markers. Furthermore, the initial scale down 1/8 worked as the initialization and most of the convergence occurred at the scale 1/4. The finals steps were a refinement process.

We varied the parameters of registration such as: iteration, multi-resolution levels, gradient step and radius neighbor for CC. We analyzed the convergence and chose gradient step as 0.1, radius 4 in cross correlation, 4 levels of resolution with scales [8,4,2,1] and iterations per level [120,100,90,60]. We observed that the algorithm converged properly for all the patients after parametrization.

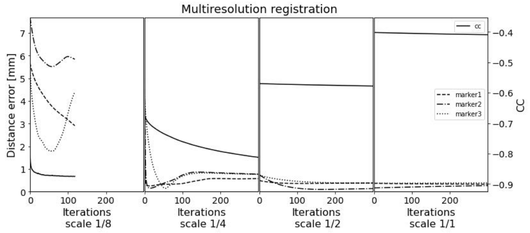

S3 Fig. Comparing distance error of fiducial markers with cross-correlation similarity metric in multi-resolution registration. The voxel intensity metric achieves convergence for the distance metric.
