# Supplementary material for: Accuracy and consistency of intensity-based deformable image registration in 4DCT for tumor motion estimation in liver radiotherapy planning
Source: PLoS One. 2022 Jul 8;17(7):e0271064. doi: 10.1371/journal.pone.0271064 (PMC9269460; doi:10.1371/journal.pone.0271064)
Supplement: S5 Appendix — (PDF) [file pone.0271064.s005.pdf]

S5 Appendix

Statistical Results

S1 Table shows the computed values of the Kolmogorov-Smirnov statistic on 2 samples. This is a two-sided test for the null hypothesis that 2 independent samples are drawn from the same continuous distribution. The test is applied to the distribution of registration errors for all the patients, comparing the results per phase obtained with the different methods. The compared methods are the reference versus sequential registration, and the inpainted versus the original images with reference registration.

S1 Table. Summary of Kolmogorov-Smirnov statistical tests. Comparisons are made for reference versus sequential registration methods, and the inpainted versus original images (reference registration). The p-values are higher than 0.05 which cannot reject the null hypothesis.

	Phase	00	10	20	30	40	50	60	70	80	90
Reference vs sequential	Statistic	0.292	0.333	0.333	0.167	0.0	0.0	0.0	0.250	0.208	0.167
	P-value	0.263	0.140	0.140	0.902	1.0	1.0	1.0	0.449	0.686	0.902
Original vs inpainted	Statistic	0.167	0.333	0.375	0.167	0.250	0.0	0.208	0.125	0.250	0.167
	P-value	0.902	0.140	0.068	0.902	0.449	1.0	0.686	0.994	0.449	0.902
