# Supplementary material for: Accuracy and consistency of intensity-based deformable image registration in 4DCT for tumor motion estimation in liver radiotherapy planning
Source: PLoS One. 2022 Jul 8;17(7):e0271064. doi: 10.1371/journal.pone.0271064 (PMC9269460; doi:10.1371/journal.pone.0271064)
Supplement: S6 Appendix — (PDF) [file pone.0271064.s006.pdf]

S6 Appendix

Registration Errors per Patient

The registration errors were comprised in a single plot for all patients. For expanding this information the following figures offer patient specifics of registration errors (see S4 Fig) and registration errors of inpainting (see S5 Fig).

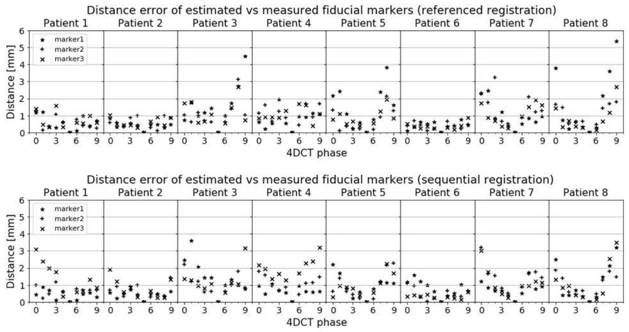

S4 Fig. Registration errors per patient. Top panel relates to refence registration and botton panel relates to sequential registration.

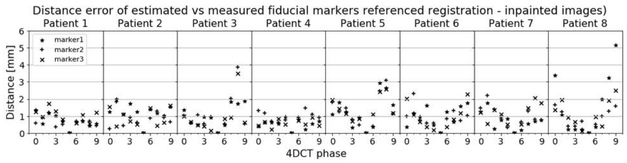

S5 Fig. Registration errors with inpainting per patient. Only referenced registration is applied for the inpainted images.
