# Supplementary material for: Accuracy and consistency of intensity-based deformable image registration in 4DCT for tumor motion estimation in liver radiotherapy planning
Source: PLoS One. 2022 Jul 8;17(7):e0271064. doi: 10.1371/journal.pone.0271064 (PMC9269460; doi:10.1371/journal.pone.0271064)
Supplement: S7 Appendix — (PDF) [file pone.0271064.s007.pdf]

S7 Appendix

Complementary Metric

In our study, we evaluated another metric to test registration errors. We exclude this from the paper for simplicity reasons. The complementary metric was defined as the relative motion discrepancy. The metric compare the GTV COM and the fiducial markers. The fiducial markers are the ground truth. The GTV COM position was calculated for every image based on the transformed GTV of the reference image. For both GTV COM and fiducial markers, the respective reference positions are substracted, i.e. the same zero coordinate point at the reference. After this, the relative motion discrepancy was then the distance between the GTV COM and the fiducial markers in the other respiratory phases of the 4DCT. This was defined as:

$$m \equiv \| \, (x_{f,j} - x_{f,\mathrm{ref}}) - (\varphi_j \circ x_{\mathrm{GTV},j} - x_{\mathrm{GTV,ref}}) \, \| \tag{7}$$

The relative motion discrepancy comprised results are shown in S6 Fig. Individual patients results are shown in S7 Fig. The same patern of high distance variations in patient 3, 6 and 7 where the fiducial markers have variations is observed. The relative motion discrepancy between GTV COM and fiducial markers had a maximum mean of 2.0mm and 1.9mm for the referenced registration and the sequential registration respectively. The maximum median values are 1.8mm for both reference and sequential registration. Similar to the registration error, no statistically significant differences between the sequential and referenced registration methods was found.

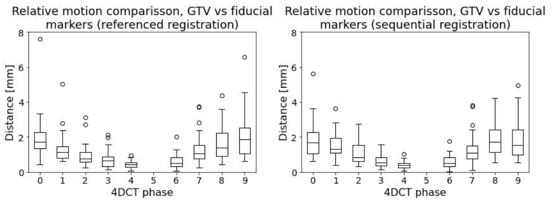

S6 Fig. Relative motion discrepancy of GTV COM compared to the relative motion of the fiducial markers. The distance metric is comprised for all the patients computed with the reference (left plot) and the sequential registration (right plot). Relative motion refers to having as zero coordinates the positions of 50% phase.

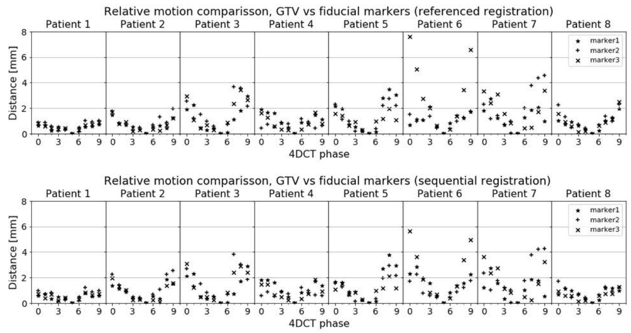

S7 Fig. Relative motion discrepancy per patient of GTV COM to the relative motion of the fiducial markers. Top panel relates to refence registration and botton panel relates to sequential registration.
